# Antinociceptive activity of the *Caesalpinia eriostachys* Benth. ethanolic extract, fractions, and isolated compounds in mice

**DOI:** 10.1002/fsn3.2846

**Published:** 2022-04-07

**Authors:** Hyun‐Yong Kim, Hee Jung Lee, Guanglei Zuo, Seung Hwan Hwang, Jeong Seok Park, Jae Seung Hong, Kang Hyuk Kim, Silvia Soto Montero, Dong‐Keun Yi, Jeong Tae Lee, Hong‐Won Suh, Soon Sung Lim

**Affiliations:** ^1^ 26727 Department of Food Science and Nutrition College of Natural Sciences Hallym University Chuncheon Korea; ^2^ 26727 Department of Pharmacology College of Medicine Hallym University Chuncheon Korea; ^3^ 26727 Department of Physical Education College of Natural Sciences Hallym University Chuncheon Korea; ^4^ Bioprospecting Research Unit National Biodiversity Institute Heredia Costa Rica; ^5^ 54679 International Biological Material Research Center Korea Research Institute of Bioscience and Biotechnology Daejeon Republic of Korea; ^6^ 26727 Department of Chemistry and Institute of Applied Chemistry Hallym University Chuncheon Republic of Korea; ^7^ 26727 Institute of Korean Nutrition Hallym University Chuncheon Republic of Korea

**Keywords:** agathisflavone, antinociceptive effect, *Caesalpinia eriostachys* Benth, pain, phytochemical analysis

## Abstract

*Caesalpinia eriostachys* Benth. (CE) is native to the Mexico and multiple effects have been observed from several plants belonging to the same family. CE was subjected to extraction with 95% ethanol, and the components were isolated through column chromatography. The structure of the compound was elucidated based on nuclear magnetic resonance (NMR) spectral data, electron ionization–mass (EI‐MS) spectroscopy, and liquid chromatography–mass (LC‐MS) spectroscopy. In vivo antinociceptive studies were conducted using writhing, 5% formalin, tail‐flick, hot‐plate, and von Frey filament tests. The ethanolic extract showed a significant effect in the acetic acid‐induced pain model and nociceptive behavior in the formalin model (second phase). In hot‐plate test and tail‐flick test, the results showed no difference compared to the control group. The results suggest that the ethanolic extract may act peripherally to reduce pain. In the streptozotocin (STZ)‐induced pain model, the ethanolic extract showed significant effect in the von Frey test model. The *n*‐Hex (Hexane) and MC (Methylene chloride) fractions and isolated compounds, ellagic acid and agathisflavone, showed increased effect. Based on these results, we confirmed that the CE ethanolic extract and their compounds, ellagic acid and agathisflavone, have antinociceptive effect on diabetes mellitus‐induced pain. Furthermore, the results of this study might be valuable for identifying compounds with antinociceptive activity from natural products.

## Graphical abstract

The ethanolic extract of *Caesalpinia eriostachys* Benth. showed significant effect in von Frey filament test. The ethanolic extract of *Caesalpinia eriostachys* Benth. (CE) showed a significant antinociceptive effect and isolated compounds, ellagic acid and agathisflavone, showed increased effect. Based on these results, the antinociceptive effect of the CE ethanolic extract and compounds was confirmed for diabetes mellitus‐induced pain.

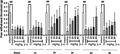

## 1 INTRODUCTION

Pain is a major health problem and one of the most prevalent conditions limiting productivity and diminishing the quality of life (Higgs et al., 2013). Pain has a physiological component called nociception, which is the process by which intense thermal, mechanical, or chemical stimuli are detected by a subpopulation called nociceptors (Regalado et al., 2017). Pain is a symptom associated with several disorders, including inflammation, and it requires analgesic treatment (Fongang et al., 2017). Nonsteroidal anti‐inflammatory drugs and opioids are widely used in pain therapy, but prolonged use of these drugs results in side effects, such as gastrointestinal damage, cardiovascular complications, tolerance, and physical and psychological dependence (Saldanha et al., 2017). For these reasons, recently, there is growing interest in the use of herbal medicinal products and the search for sources of new drugs (Fongang et al., 2017). For a long time, medicinal plants have been traditionally used for relief from disease, and certain compounds derived from plants present significant analgesic properties (S. Gorzalczany et al., 2011). *Caesalpinia* species are distributed throughout the tropic and subtropics, and used folkloric medicine to treat various diseases (Chan et al., 2018). *Caesalpinia eriostachys* is native species included in the *Caesalpinia* genus (Fabaceae), commonly used in the west of Mexico as an ornamental tree and forage (Pamatz‐Bolaños et al., 2018). *Caesalpinia* plants belonging to the Caesalpiniaceae family have shown various effects, including hepato‐protective effect (Kumar et al., 2010), anti‐inflammatory effect (Kale et al., 2010), and antitumor activity (Gupta et al., 2004). Furthermore, some of the chemical constituents in the plants of the *Caesalpinia* family have also shown beneficial effects; flavones, isoflavones, and chalcone, isolated from *Caesalpinia pulcherrima* showed anti‐inflammatory activity (Rao et al., 2005), and brazilin isolated from *Caesalpinia sappan* L. was responsible for anti‐inflammatory and wound‐healing effects (Tewtrakul et al., 2015). However, no previous studies have evaluated the antinociceptive potential of *Caesalpinia eriostachys* Benth. (CE). In this study, we confirmed the antinociceptive effects of the crude extract of CE and its individual compounds, indicating the potential for the application as new strategies for pain treatment.

## 2 MATERIALS AND METHODS

### 2.1 Chemical and reagents

All chemicals were analytical grade. Methanol (MeOH) was acquired from J. T. Baker Chemical Company (Phillipsburg, New Jersey, USA), and formic acid was purchased from Sigma Chemical Company (St. Louis, MO, USA). Ethanol (EtOH), hexane (*n*‐Hex), methylene chloride (MC), ethyl acetate (EtOAc), and butanol (*n*‐BuOH) were purchased from Samchun Chemicals (Seoul, Korea). Acetic acid and formalin were purchased from Deajung chemicals (Siheung, Gyeonggi‐do, Korea).

### 2.2 Plant material

CE was harvested at Santa Rosa national park, Guanacaste conservation area, Guanacaste, Costa Rica in August 2017 (the authorization by the Technical Office of the National Commission for Biodiversity Management, CONAGEBIO, Costa Rica) by National Biodiversity Institute (INBio) and the voucher specimen (KRIB 0079756) was deposited at the herbarium of International Biological Material Research Center, Korea Research Institute of Bioscience and Biotechnology (KRIBB), Republic of Korea.

### 2.3 Extract and isolation

**FIGURE 1:**
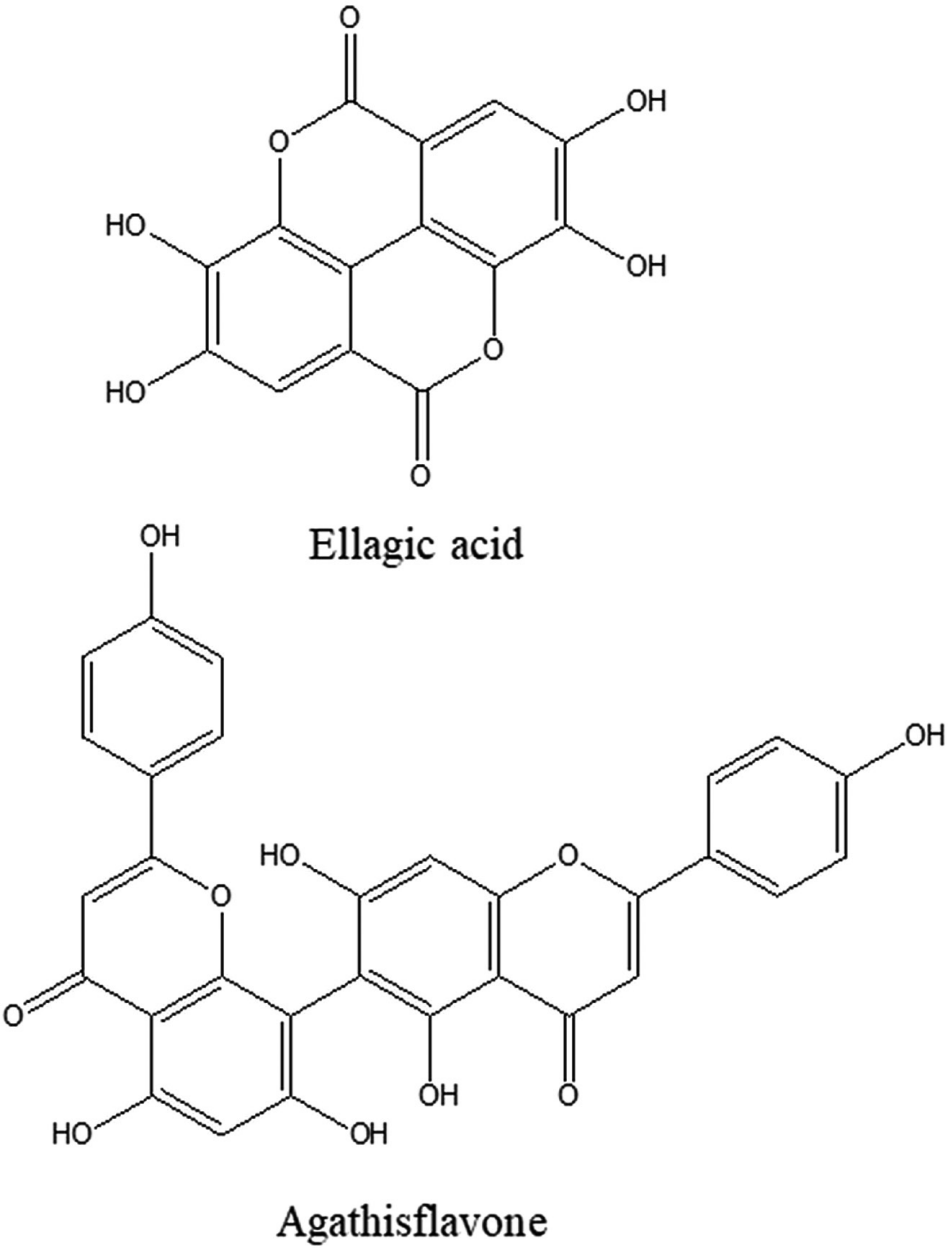
Chemical structure of ellagic acid and agathisflavon

CE was subjected to extraction with 95% ethanol (EtOH) for 3 days at room temperature. The filtrate was concentrated to dryness in vacuo at 37°C. The major components from CE extract were isolated by column chromatography. The CE ethanolic extract was systemically partitioned into five fractions: hexane (*n*‐Hex), methylene chloride (MC), ethyl acetate (EtOAc), butanol (*n*‐BuOH), and water. Among the fractions, the *n*‐Hex fraction was purified using Sephadex LH‐20 (100% MeOH) to furnish 22 fractions: Fr.1–Fr.22. Compound 1 (33 mg) and compound 2 (19.2 mg) were obtained from Fr.11 and Fr.15, respectively. The structures of the compounds isolated in this manner were elucidated based on 1D and 2D (heteronuclear single quantum coherence and heteronuclear multiple bond correlation; HSQC and HMBC) nuclear magnetic resonance (NMR) spectral data, electron ionization–mass spectroscopy (EI‐MS), and liquid chromatography–mass spectroscopy/mass spectroscopy (LC‐MS/MS) by comparison with published data (Ajileye et al., 2015; Lee et al., 2005) (Figure 1).

#### 2.3.1 Compound (1): Ellagic acid

UV λ_max_ nm: 254, 300, 366 nm, IR v_max_ cm^−1^:3557.5, 3476, and 1698.5. LC‐MS/MS m/z 300.6, 285.1, 275.2, and 257.2 [M‐H]^‐^. ^1^H‐NMR (600 MHz, CD_3_OD, CD_3_COCD_3,_ δ_H_) δ 7.55 (2H, s, H‐5/H‐5’).

#### 2.3.2 Compound (2): Agathisflavone

UV λ_max_ nm: 229, 298 nm, IR v_max_ cm^−1^:3350, 1650, 1605, 1520, and 1500. EI‐MS m/z 538 [M + H]^+^. ^1^H‐NMR (400MHz, CD_3_OD, δ_H_) δ 6.36 (1H, s, H‐6’’), δ 6.60 (1H, s, H‐3’’), δ 6.68 (1H, s, H‐3), δ 6.70 (1H, s, H‐8), δ 6.76 (2H, d, J = 8.4, H‐3’’’, H‐5’’’), δ 6.96 (2H, d, J = 8.4, H‐3’, H‐5’), δ 7.56 (2H, d, J = 8.4, H‐2’’’, H‐6’’’), δ 7.93 (2H, d, J = 8, H‐2’, H‐6’); ^13^C‐NMR (600MHz, CD_3_OD, δc) δ 94.80 (C‐8), δ 100.04 (C‐6’’), δ 100.51 (C‐8’’), δ 103.58 (C‐3’’), δ 104.11 (C‐3), δ 104.95 (C‐6), δ 105.47 (C‐10), δ 105.69 (C‐10’’), δ 117.02 (C‐3’, C‐5’), δ 117.21 (C‐3’’’, C‐5’’’), δ 123.44 (C‐1’), δ 123.54 (C‐1’’’), δ 129.37 (C‐2’’’, C‐6’’’), δ 129.67 (C‐2’, C‐6’), δ 157.10 (C‐9’’), δ 159.21 (C‐9), δ 161.55 (C‐5), δ 162.69 (C‐5’’), δ 162.77(C‐4’’’), δ 162.94(C‐4’), δ 164.27 (C‐7’’), δ 164.53 (C‐7), δ 166.26 (C‐2’’), δ 166.41 (C‐2), δ 184.15(C‐4’’), δ 184.47(C‐4).

### 2.4 Phytochemical analysis: high‐performance liquid chromatography (HPLC)

Chromatographic analysis of the compounds from the CE extract was performed using an HPLC system (Thermo Finnigan Surveyor, San Jose, CA, USA). The separations were carried out under gradient conditions using an Agilent (Santa Clara, CA, USA) ZORBAX column (4.6 μm × 25 cm, with 3.5 μm particle size) at 37°C. The mobile phase composed of water containing 0.1% formic acid (A) and MeOH (B), according to the following elution program: 5%–100% B (0–40 min); 100% B (40–43 min); 100%–5% B (43–45 min); and 5% B (45–50 min). Peaks were detected using a Ultraviolet detector at 280 nm. The flow rate was 0.7 ml/min, and the injection volume was 10 μL.

### 2.5 Animals

#### 2.5.1 Experimental animals

Male ICR mice (6 weeks of age), weighing 25–30 g at the beginning of experiments (Koatech, Seoul, Korea), were used. The mice were housed five per one cage in a room that was maintained at 22 ± 1°C with an alternating 12‐h light–dark cycles. Food and water were available ad libitum. The animals were allowed to adapt at least 2 h before testing and were only use one time. For reducing variation, all experiments were performed during the light phase of the cycle (10:00 ~ 17:00). The experiment was approved by the Hallym University Institutional Animal Care and Use Committees (Registration Number: Hallym 2018–71), according to the ‘Guide for Care and Use of Laboratory Animals’ published by the National Institutes of Health and the ethical guidelines of the International Association for the Study of Pain. No mice died and exhaustion was not observed during experiment. After completion of all experiments, all mouse groups were euthanized with 2,2,2‐tribromoethanol (Sigma Aldrich, Saint Louis, Missouri, USA) and 2‐methyl‐2‐butanol (Sigma Aldrich, Saint Louis, Missouri, USA).

#### 2.5.2 Streptozotocin (STZ)‐induced diabetic mice

Diabetes was induced in mice via a single intraperitoneal injection of STZ (75 ~ 80 mg/kg in citrate buffer, pH 4.5). STZ was injected for 3 days. Normal groups received the buffer alone. On the fourth day after STZ administration, glucose levels were measured using a glucometer (ACCU‐CHEKPerforma, Roche Diagnostics, Mannheim, Germany). Animals with nonfasting blood glucose concentration above 250 mg/dl were considered diabetic and used in the current study. After one week, five mice each were treated with normal saline (5 ml/kg, b.w., p.o.), ethanolic extract (10–80 mg/kg, b.w., p.o.), fractions (40 mg/kg, b.w., p.o.), and compound (40 mg/kg, b.w., p.o.).

#### 2.5.3 In vivo antinociceptive studies

##### Acetic acid‐induced writhing test

For the visceral pain model, 1% acetic acid was used to the mice model (Koster et al., 1959). Four groups of five mice each were pretreated orally (p.o.) with normal saline (5 ml/kg, b.w., p.o.) and ethanolic extract (5, 10, and 20 mg/kg, b.w., p.o.). About 30 min after the administration, 1% acetic acid was injected intraperitoneally (i.p.) to mice. The number of writhing responses was counted for 30 min and began 5 min after acetic acid injection.

##### Formalin test

For the formalin pain model (S. Hunskaar et al., 1985; Steinar Hunskaar & Hole, 1987), four groups of five mice each were treated orally with normal saline (5 ml/kg, b.w., p.o.) and ethanolic extract (5, 10, and 20 mg/kg, b.w., p.o.). After 30 min, 10 µl of 5% formalin was injected subcutaneously into the plantar surface of the left hind paw of each animal using a 50‐µL Hamilton syringe. The time that the animal spent licking or biting its paws was measured during the first (0–5 min) and the second (20–40 min) phases of the test.

##### Tail‐flick and hot‐plate tests

Antinociception was determined by the tail‐flick (D'Amour & Smith, 1941) and the hot‐plate paw‐licking tests (Eddy & Leimbach, 1953). For the measurement of the tail‐flick latency, mice were held with the tail positioned in the apparatus (EMDIE Instrument Co., Maidens, VA, USA, Model TF6), and the tail‐flick response was elicited by applying radiant heat to the dorsal surface of the tail. The test was repeated three times to obtain the mean value of the tail‐flick latency. The tail of the mouse not exposed to the heat for more than 20 s to avoid burns. Five mice each were pretreated orally (p.o.) with normal saline (5 ml/kg, b.w., p.o.) and ethanolic extract (10, 20, 40, or 80 mg/kg, b.w., p.o.), 30 min before initiating the algesic stimulation. For the hot‐plate test, the mice were individually placed on the 55°C hot‐plate apparatus (Itic Life Science, Woodland Hills, CA, USA, Model 39 Hot Plate); next, the reaction time was measured, starting from the placement of the mouse on the hot‐plate to the time of licking the front paw. The latency to the paws was the reaction time. The cut‐off time was set at 20 s to avoid damage to the skin. Five mice each were pretreated orally (p.o.) with ethanolic extract (10, 20, 40, or 80 mg/kg, b.w., p.o.) or normal saline (5 ml/kg, b.w., p.o.), 30 min before initiating the algesic stimulation.

##### von Frey filament tests

For the von Frey test, the number of animals used in the experiment was five in each group. The tactile withdrawal threshold of mice was measured by using the von Frey method (Bonin et al., 2014; Chaplan et al., 1994). Mice were placed individually in wire‐mesh floor cages (8 cm ×8 cm) to allow insertion of the mechanical probe from below against the plantar surface of the hind paw. The animals were allowed to habituate to the testing chambers for at least 1 h before the first measurement. After an acclimation period, we stimulated the plantar surface of the left hind paw vertically with a series of von Frey filaments (North Coast Medical, Inc., Gilroy, CA, USA) with logarithmically increasing stiffness. The filament was bent for 5 s to the central plantar surface with a sufficient force, and brisk withdrawal or paw flinching was considered as a positive response. The test of tactile withdrawal threshold was repeated two times in each mouse, and the mean value was calculated. Mechanical hyperalgesia was assessed from 1–5 h after the oral administration of normal saline (5 ml/kg, b.w., p.o.), ethanolic extract (10 ~ 80 mg/kg, b.w., p.o.), and from 1–4 h after the administration of fractions (40 mg/kg, b.w., p.o.) and compounds (40 mg/kg, b.w., p.o.).

### 2.6 Statistical analysis

All values are expressed as the mean ± *SD*. Comparison between groups was performed using Student's *t*‐test. A *p* < .05 was considered statistically significant.

## 3 RESULTS

### 3.1 Phytochemical analysis

**FIGURE 2:**
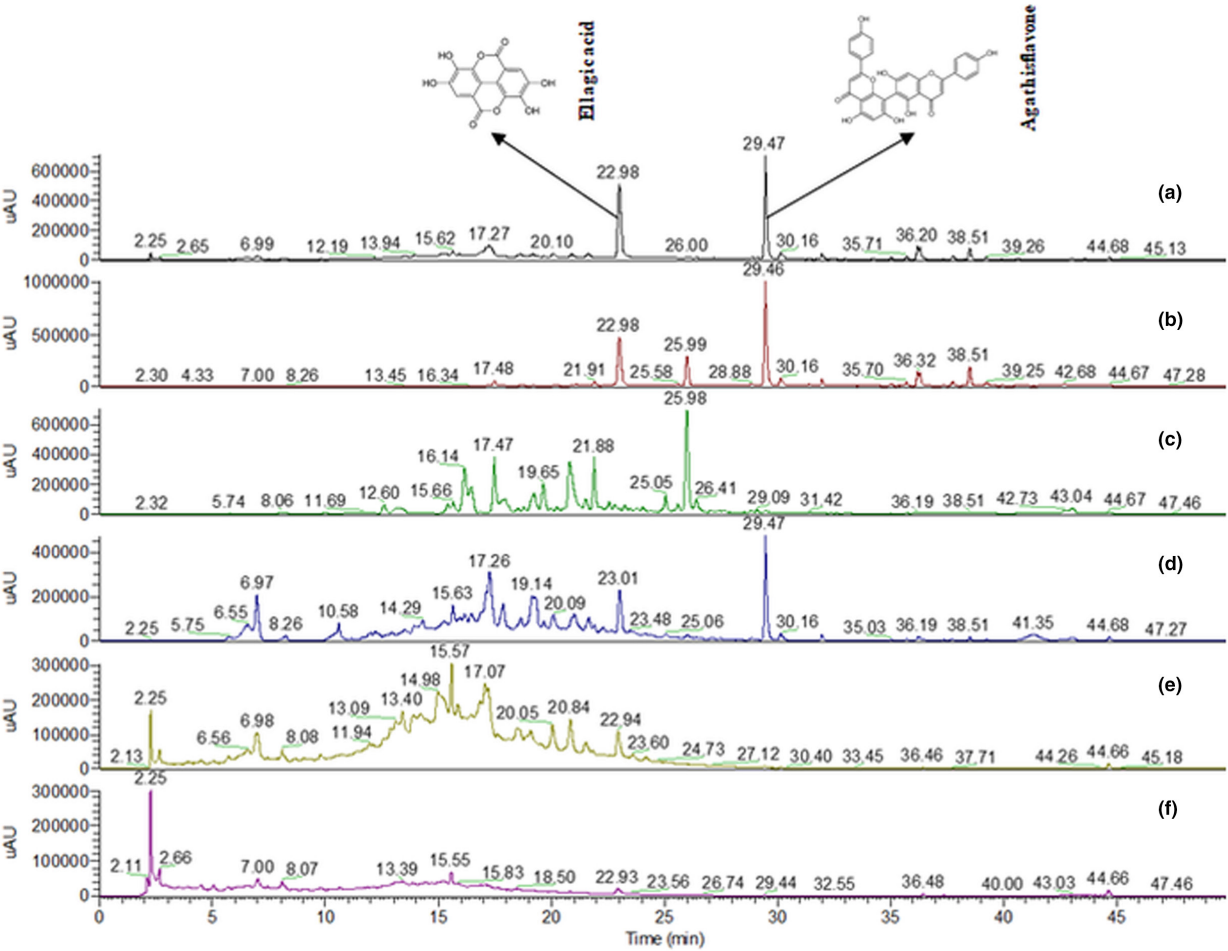
Chromatogram of the CE extract and fractions. The groups are shown as follows: (a) CE ethanolic extract and fractions [(b) Hexane (*n*‐Hex), (c) Methylene chloride (MC), (d) Ethyl acetate (EtOAc), (e) Butanol (*n*‐BuOH), and (f) water]

HPLC analysis of the CE ethanolic extract showed the presence of the compounds ellagic acid (R_T_: 22.98 min) and agathisflavone (R_T_: 29.47 min). The fingerprint chromatogram is shown in Figure 2.

### 3.2 Antinociceptive effect of the CE extract in the tail‐flick and hot‐plate tests

**FIGURE 3:**
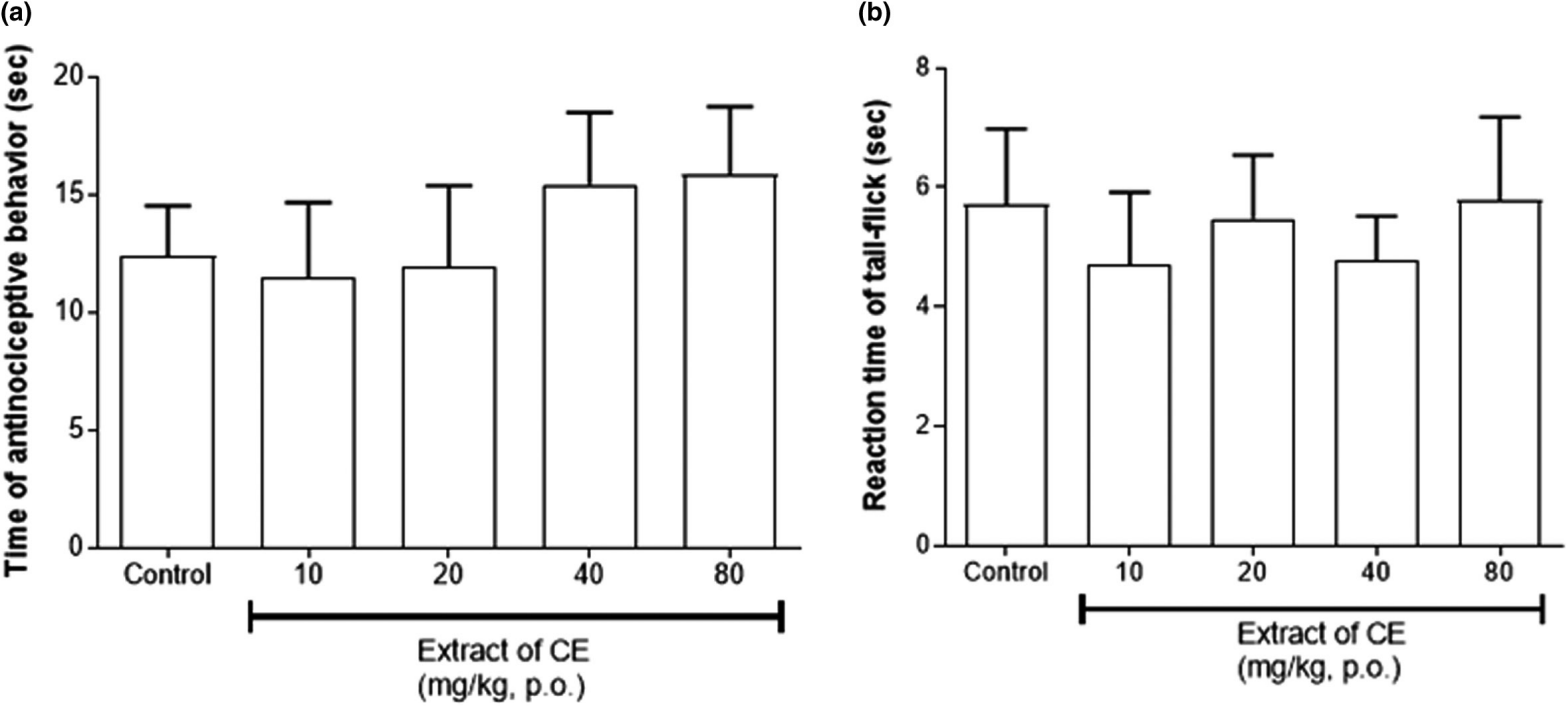
Effect of CE ethanolic extract in the hot‐plate test (a) and tail‐flick test (b). Data are expressed as mean ± *SD*; *n* = 5 mice per group

The result presented in Figure 3 shows that doses of the CE extract at 10, 20, 40, and 80 mg/kg were used for the hot‐plate test and tail‐flicking test. In the hot‐plate test, all CE extract treated group showed similar antinociceptive behavior compared to the control group (Figure 3a). Compared to the previous experiment, the data of tail‐flicking test also showed same results (Figure 3b).

### 3.3 Antinociceptive effect of CE extract in the acetic acid‐induced abdominal writhing test and 5% formalin‐induced nociception

**FIGURE 4:**
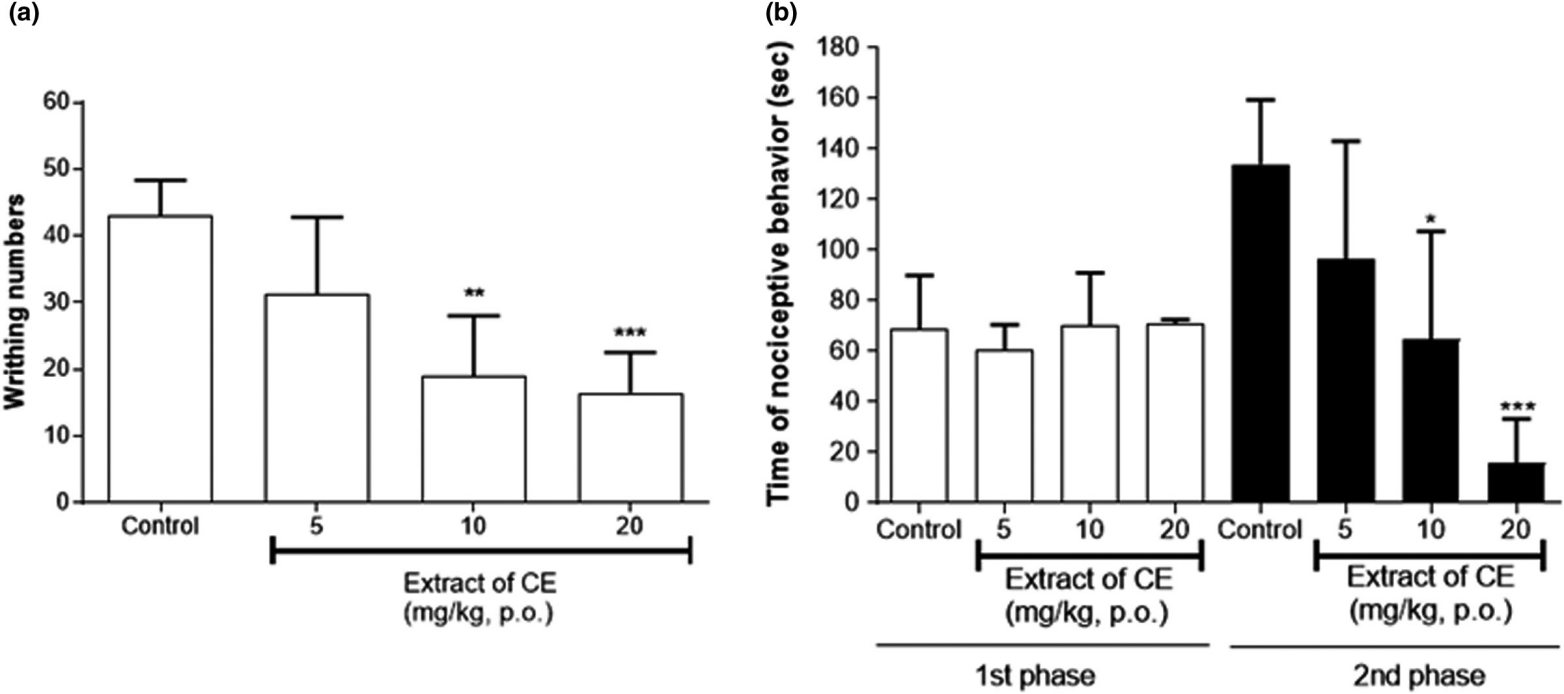
Effect of ethanolic extract of CE in the acetic acid‐induced abdominal writhing test (a) and formalin test (b) in mice. Data are expressed as mean ± *SD*; *n* = 5 mice per group (^*^ *p* < .05, ^**^ *p* < .01, ^***^ *p* < .001 versus. control group)

The result presented in Figure 4a shows that the CE extract (doses of 5 mg/kg, 10 mg/kg, and 20 mg/kg) significantly inhibited the acetic acid‐induced nociception compared to control group at doses of 10 and 20 mg/kg, respectively. As illustrated in Figure 4b, the CE extract at same doses (5 mg/kg, 10 mg/kg, and 20 mg/kg) did not show significant reduction in the nociceptive behavior time in the first phase. But, in the second phase, the CE extract reduced the nociceptive behavior time at same doses. Notably, significant reduction in nociceptive behavior time was observed in the second phase at the dose of 10 mg/kg and 20 mg/kg compared to that in the control group.

### 3.4 Antinociceptive effect of extract, fractions, and compounds isolated from CE on mechanical pain thresholds in STZ‐induced pain model

**FIGURE 5:**
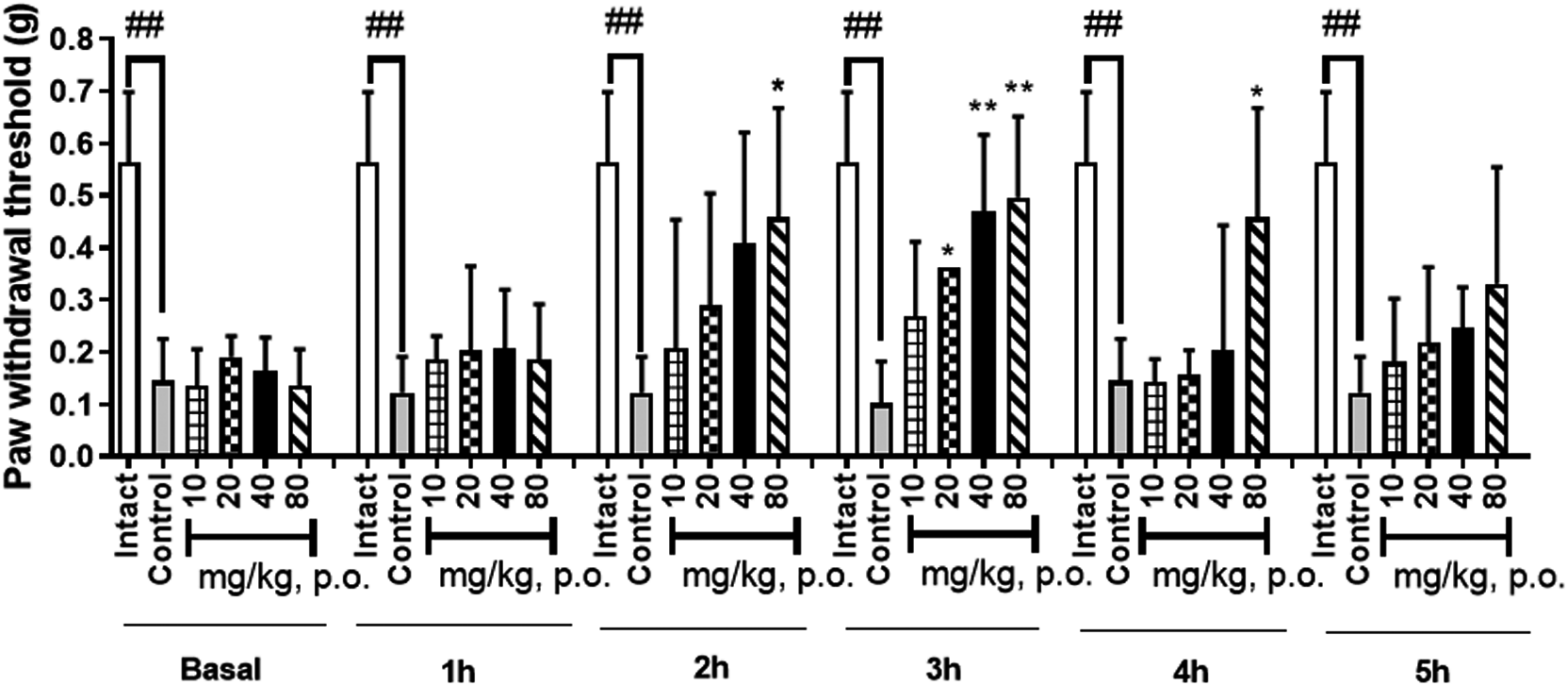
Effect of CE ethanolic extract, according to the von Frey filament test, on the streptozotocin‐induced mouse model of diabetic peripheral neuropathic pain. Data are expressed as mean ± *SD*; *n* = 5 mice per group (^##^ *p* < .01 versus. intact group; ^*^ *p* < .05, ^**^ *p* < .01 versus. control group)

**FIGURE 6:**
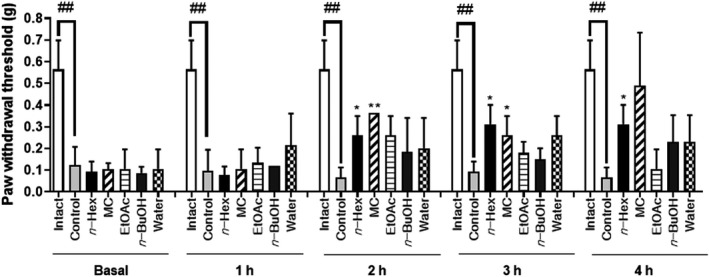
Effect of CE ethanolic extract fractionations, according to the von Frey filament test, on the streptozotocin‐induced mouse model of diabetic peripheral neuropathic pain. Data are expressed as mean ± *SD*; *n* = 5 mice per group (^##^ *p* < .01 versus. intact group; ^*^ *p* < .05, ^**^ *p* < .001 versus. control group)

**FIGURE 7:**
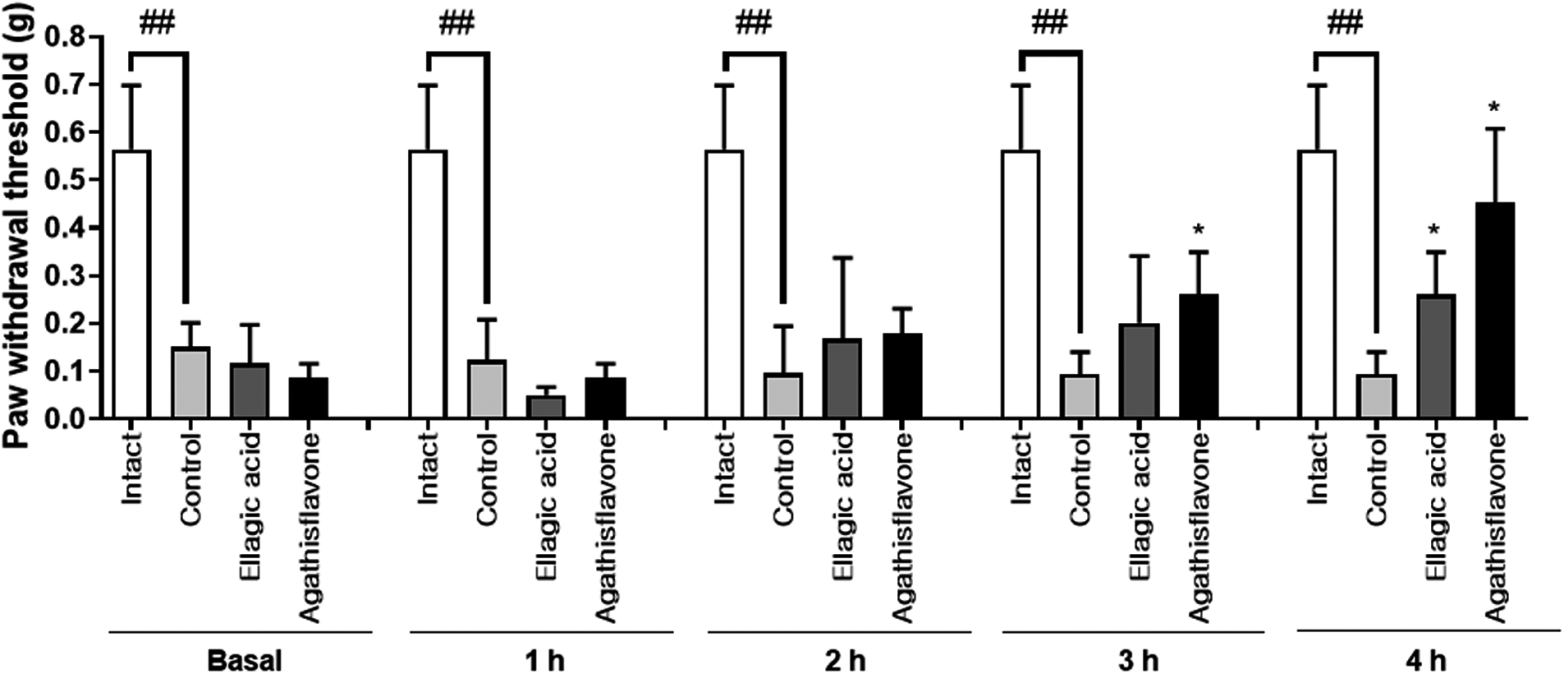
Effect of isolated compounds from CE ethanolic extract, according to the von Frey filament test, on the streptozotocin‐induced mouse model of diabetic peripheral neuropathic pain. Data are expressed as mean ± *SD*; *n* = 5 mice per group (^##^ *p* < .01 versus. intact group; ^*^ *p* < .05 versus. control group)

As shown in Figure 5, the extract of CE was potent enough to be highly effective in pain models. Among the concentrations tested, the group that were administered 20–80 mg/kg CE extract showed significantly lower nociception level than the control group at the 3‐h time point; at 2‐ and 4‐h time points, the 80 mg/kg of CE extract appeared to show increased antinociceptive effect. After confirming the antinociceptive effect of CE extract from the writhing test, formalin test (second phase), and the von Frey model, we proceeded to confirm the activity of the CE fractions at a dose of 40 mg/kg in the STZ‐induced pain model. Five fractions from the CE extract were used for confirmation of activity; the *n*‐Hex and MC fractions showed increased antinociceptive effect from 2 h to 4 h (Figure 6). The *n*‐Hex fraction showed an increase in antinociceptive effect with the passage of time and presented major peaks of the CE extract; we isolated ellagic acid and agathisflavone from this fraction. The compounds isolated from the *n*‐Hex fraction, ellagic acid and agathisflavone, were also tested via the von Frey test at a dose of 40 mg/kg for further characterization of these compounds. Both ellagic acid and agathisflavone showed an increase in antinociceptive effect with the passage of time in the STZ‐induced diabetic mice (Figure 7).

## 4 DISCUSSION

In the present study, we evaluated the antinociceptive effect of the CE ethanolic extract and two isolated compounds, ellagic acid and agathisflavone, for the development of new strategies for the treatment of pain.

Several studies were processed about compounds with antinociceptive effect in natural products, including *Urtica circularis* (S Gorzalczany et al., 2011), *Bryophyllum pinnatum* (Crassulaceae) (Ojewole, 2005), and *Cenostigma macrophyllum* (LEGUMINOSAE) (Alves et al., 2012). In case of mechanism of pain, AMPK activation signaling can effect to several pathologies including chronic neuropathic pain, and metformin directly decreases pain by decreasing the activity of mTORC1 and MAPK signaling in nociceptors (Baeza‐Flores et al., 2020). The antinociceptive activities of the extract were evaluated using the acetic acid‐induced writhing test, 5% formalin‐induced pain model, hot‐plate test, and tail‐flicking test. The hot‐plate method is suitable for the evaluation for central nociceptive activity (Sharma et al., 2019), and the tail‐flicking test measures spinal nociceptive response latencies to a thermal stimulus such as a hot plate (Lima Cavendish et al., 2015). Both tests are simple and commonly used to study analgesic efficiency and less sensitive to non‐opioid analgesics (Karna et al., 2019). In the hot‐plate test, doses of 40 mg/kg and 80 mg/kg showed slightly increased antinociceptive behavior compared to the control, but not significantly different. The tail‐flicking test also showed no significant difference in the treatment groups compared to the control group (*p* >.05). The acetic acid‐induced writhing test is a visceral model for evaluating peripheral antinociceptive activity, and the pain in this model is generated via endogenous mediators (Wang et al., 2014). The CE extract showed a significant decrease in the number of writhing responses in the acetic acid‐induced pain model at doses of 10 and 20 mg/kg, suggesting that the CE extract may act to reduce peripheral pain in mice. The formalin‐induced pain test was used to evaluate neurogenic pain responses in mice; first phase has been considered to reflect direct chemical stimulation of C‐fibers (neurogenic pain) and second phase is dependent on peripheral tissue inflammation (inflammatory pain) (Tjølsen et al., 1992). Additionally, the first phase is induced by the ion channel TRPA 1, and the second phase is effected by a combination of peripheral input and sensitization of spinal cord neurons (Lima Cavendish et al., 2015). The CE extract showed a similar effect as the control in the first phase; however, in the second phase, it significantly reduced nociceptive behavior at doses of 10 and 20 mg/kg. Based on these results, it was confirmed that treatment with the CE extract showed an antinociceptive effect in the mouse model of peripheral and neurogenic pain, but not in the model of central and spinal nociception. The results of four tests suggest that CE may act peripherally to reduce pain.

After evaluating the effect of the CE extract through four pain tests, we confirmed that treatment with the extract, fractions, and compounds of the CE extract reduced the pain threshold, as evaluated by the von Frey filament test in STZ‐induced diabetic mice. Diabetes induced by STZ causes glial activation, glutamate toxicity, and neuronal death, which could induce necrotic and apoptotic cell death (Chiu et al., 2016). STZ‐induced diabetes is a commonly used experimental model of neuropathic pain (Huang et al., 2016). When we evaluated the antinociceptive effect of the CE extract via the von Frey test, a significantly lower nociception was observed at doses of 20, 40, and 80 mg/kg, compared with the nociception observed for the control group at the 3‐h time point. Although the effect was lower after 3 h, this result suggested that the CE extract exhibits a dose‐dependent increase in antinociceptive activity, according to using higher concentrations. The activity of the CE fractions was confirmed at a dose of 40 mg/kg in the STZ‐induced pain model. Among the five fractions, the *n*‐Hex (2–4 h) and MC (2–3 h) fractions showed antinociceptive effect at the time point. The *n*‐Hex fraction was selected for isolation of the active compounds, ellagic acid and agathisflavone, because this fraction contained the major peak of the CE extract, according to the previous result observed in the STZ‐induced pain model.

The isolated compounds, ellagic acid and agathisflavone, were tested at a dose of 40 mg/kg, and both showed increased effect with the passage of time in STZ‐induced diabetic mice. Mansouri et.al (Taghi Mansouri et al., 2013) confirmed the dose‐related antinociceptive effect of ellagic acid, involving mediation by the opioidergic system and l‐arginine–NO–cGMP–ATP sensitive K^+^ channels pathway, and ellagic acid showed antinociceptive action in the acetic acid‐induced mouse model of pain (Mansouri et al., 2015). In the case of agathisflavone, a fraction of *Cenostigma macrophyllum* Tul. Var. *acuminate* Teles Freire that contained agathisflavone showed antinociceptive action in rodents (Cavalcanti et al., 2017), neuroprotective and anti‐inflammatory effects (Dos Santos Souza et al., 2018), and decrease in oxidative stress in the brain (Dumitru et al., 2019). However, so far, there is no available research on the antinociceptive activity of agathisflavone as an isolated compound in various pain models. For this reason, the present study supports the use of CE as a potential analgesic antinociceptive agent. Based on the results, we conclude that CE is a novel antinociceptive agent and can be used as a therapeutic option in the treatment of neuropathic pain associated with diabetes mellitus.

## 5 CONCLUSIONS

In summary, our data demonstrated that administration of the ethanolic extract, fractions, and compounds of CE exhibits antinociceptive activity according to different pain tests induced by acetic acid, a hot plate, 5% formalin, and STZ. The CE extract showed a significant decrease in the number of writhing responses in the acetic acid‐induced pain model and in nociceptive behavior in the second phase of the formalin model. In the hot‐plate test, the CE groups showed increased antinociceptive behavior compared to the control group, but this effect was not significant. The results of the tail‐flicking test also showed no significant difference between the treatment groups and the control group. In the STZ‐induced pain model, the *n*‐Hex and MC fractions of the CE extract showed an effect at the 2‐to 4‐h time point, and two compounds from the *n*‐Hex fraction, ellagic acid and agathisflavone, showed increased effect with the passage of time. Based on these results, the antinociceptive effect of the CE extract, ellagic acid, and agathisflavone was confirmed for peripheral and neurogenic pain. The results of this study might be valuable for identifying compounds with antinociceptive activity from natural products.

## CONFLICT OF INTEREST

The authors declare that they have no conflict of interest.

## ETHICAL STATEMENT

This study was approved by the Institutional Review Board of Hallym University.

## Data Availability

The datasets generated during and/or analysed during the current study are available from the corresponding author on reasonable request.

## References

[fsn32846-bib-0001] Ajileye, O. , Obuotor, E. , Akinkunmi, E. , & Aderogba, M. (2015). Isolation and characterization of antioxidant and antimicrobial compounds from *Anacardium occidentale* L. (Anacardiaceae) leaf extract. Journal of King Saud University‐Science, 27(3), 244–252. 10.1016/j.jksus.2014.12.004

[fsn32846-bib-0002] Alves, C. Q. , David, J. M. , David, J. P. , Villareal, C. F. , Soares, M. B. , Queiroz, L. P. , & Aguiar, R. M. (2012). Flavonoids and other bioactive phenolics isolated from *Cenostigma macrophyllum* (Leguminosae). Química Nova, 35(6), 1137–1140. 10.1590/S0100-40422012000600013

[fsn32846-bib-0003] Baeza‐Flores, G. D. C. , Guzmán‐Priego, C. G. , Parra‐Flores, L. I. , Murbartian, J. , Torres‐López, J. E. , & Granados‐Soto, V. (2020). Metformin: A prospective alternative for the treatment of chronic pain. Frontiers in Pharmacology, 11, 10.3389/fphar.2020.558474 PMC7538784 33178015

[fsn32846-bib-0004] Bonin, R. P. , Bories, C. , & De Koninck, Y. (2014). A simplified up‐down method (SUDO) for measuring mechanical nociception in rodents using von Frey filaments. Molecular Pain, 10, 1710–1726. 10.1186/1744-8069-10-26 PMC4020614 24739328

[fsn32846-bib-0005] Cavalcanti, M. , Fernandes, H. , Pereira, S. , Piauilino, C. , Costa, C. , Chaves, M. , Ibiapina, J. , Marques, R. , Oliveira, F. , & Almeida, F. (2017). Antinociceptive and Anti‐inflammatory Effects of the Hydroalcoholic Fraction from Leaves of *Cenostigma macrophyllum* Tul. var. *acuminata* Teles Freire (Leguminosae) in Rodents. Orthopedics and Rheumatology Open Access Journal, 8, 2. 10.19080/OROAJ.2017.08.555733

[fsn32846-bib-0006] Chan, E. W. C. , Tangah, J. , Baba, S. , Chan, H. T. , Kainuma, M. , & Inoue, T. (2018). *Caesalpinia crista*: A coastal woody climber with promising therapeutic values. Journal of Applied Pharmaceutical Science, 8(03), 133–140. 10.7324/JAPS.2018.8319

[fsn32846-bib-0007] Chaplan, S. R. , Bach, F. , Pogrel, J. , Chung, J. , & Yaksh, T. (1994). Quantitative assessment of tactile allodynia in the rat paw. Journal of Neuroscience Methods, 53(1), 55–63. 10.1016/0165-0270(94)90144-9 7990513

[fsn32846-bib-0008] Chiu, Y. C. , Liao, W. T. , Liu, C. K. , Wu, C. H. , & Lin, C. R. (2016). Reduction of spinal glycine receptor‐mediated miniature inhibitory postsynaptic currents in streptozotocin‐induced diabetic neuropathic pain. Neuroscience Letters, 611, 88–93. 10.1016/j.neulet.2015.10.072 26598022

[fsn32846-bib-0009] D'Amour, F. E. , & Smith, D. L. (1941). A method for determining loss of pain sensation. Journal of Pharmacology and Experimental Therapeutics, 72(1), 74–79.

[fsn32846-bib-0010] Dos Santos Souza, C. , Grangeiro, M. S. , Lima Pereira, E. P. , Dos Santos, C. C. , da Silva, A. B. , Sampaio, G. P. , Ribeiro Figueiredo, D. D. , David, J. M. , David, J. P. , da Silva, V. D. A. , Butt, A. M. L. , & Costa, S. (2018). Agathisflavone, a flavonoid derived from *Poincianella pyramidalis* (Tul.), enhances neuronal population and protects against glutamate excitotoxicity. Neurotoxicology, 65, 85–97. 10.1016/j.neuro.2018.02.001 29425760

[fsn32846-bib-0011] Dumitru, G. , El‐Nashar, H. A. S. , Mostafa, N. M. , Eldahshan, O. A. , Boiangiu, R. S. , Todirascu‐Ciornea, E. , Hritcu, L. , & Singab, A. N. B. (2019). Agathisflavone isolated from *Schinus polygamus* (Cav.) Cabrera leaves prevents scopolamine‐induced memory impairment and brain oxidative stress in zebrafish (*Danio rerio*). Phytomedicine, 58, 152889. 10.1016/j.phymed.2019.152889. 30901660

[fsn32846-bib-0012] Eddy, N. B. , & Leimbach, D. (1953). Synthetic analgesics. II. Dithienylbutenyl‐and dithienylbutylamines. Journal of Pharmacology and Experimental Therapeutics, 107(3), 385–393. 13035677

[fsn32846-bib-0013] Fongang, A. L. M. , Laure Nguemfo, E. , Djouatsa Nangue, Y. , Bogning Zangueu, C. , Fouokeng, Y. , Azebaze, A. G. B. , Jose Llorent‐Martinez, E. , Cordova, M. L. F. , Bertrand Dongmo, A. , & Vierling, W. (2017). Antinociceptive and anti‐inflammatory effects of the methanolic stem bark extract of Antrocaryon klaineanum Pierre (Anacardiaceae) in mice and rat. Journal of Ethnopharmacology, 203, 11–19. 10.1016/j.jep.2017.03.036 28342857

[fsn32846-bib-0014] Gorzalczany, S. , Marrassini, C. , Miño, J. , Acevedo, C. , & Ferraro, G. (2011). Antinociceptive activity of ethanolic extract and isolated compounds of *Urtica circularis* . Journal of Ethnopharmacology, 134(3), 733–738. 10.1016/j.jep.2011.01.025 21277970

[fsn32846-bib-0015] Gupta, M. , Mazumder, U. K. , Kumar, R. S. , Sivakumar, T. , & Vamsi, M. L. (2004). Antitumor activity and antioxidant status of *Caesalpinia bonducella* against Ehrlich ascites carcinoma in Swiss albino mice. Journal of Pharmacological Sciences, 94(2), 177–184. 10.1254/jphs.94.177 14978356

[fsn32846-bib-0016] Higgs, J. , Wasowski, C. , Loscalzo, L. M. , & Marder, M. (2013). In vitro binding affinities of a series of flavonoids for mu‐opioid receptors. Antinociceptive effect of the synthetic flavonoid 3,3‐dibromoflavanone in mice. Neuropharmacology, 72, 9–19. 10.1016/j.neuropharm.2013.04.020 23624290

[fsn32846-bib-0017] Huang, Q. , Chen, Y. , Gong, N. , & Wang, Y. X. (2016). Methylglyoxal mediates streptozotocin‐induced diabetic neuropathic pain via activation of the peripheral TRPA1 and Nav1.8 channels. Metabolism, 65(4), 463–474. 10.1016/j.metabol.2015.12.002 26975538

[fsn32846-bib-0018] Hunskaar, S. , Fasmer, O. B. , & Hole, K. (1985). Formalin test in mice, a useful technique for evaluating mild analgesics. Journal of Neuroscience Methods, 14(1), 69–76. 10.1016/0165-0270(85)90116-5 4033190

[fsn32846-bib-0019] Hunskaar, S. , & Hole, K. (1987). The formalin test in mice: Dissociation between inflammatory and non‐inflammatory pain. Pain, 30(1), 103–114. 10.1016/0304-3959(87)90088-1 3614974

[fsn32846-bib-0020] Kale, S. , Gajbhiye, G. , & Chaudhari, N. (2010). Anti‐inflammatory effect of petroleum ether extract of *Caesalpinia bonduc* (L.) Roxb seed kernel in rats using carrageenan‐induced paw edema. International Journal of PharmTech Research, 2(1), 750–752.

[fsn32846-bib-0021] Karna, S. R. , Kongara, K. , Singh, P. M. , Chambers, P. , & Lopez‐Villalobos, N. (2019). Evaluation of analgesic interaction between morphine, dexmedetomidine and maropitant using hot‐plate and tail‐flick tests in rats. Veterinary Anaesthesia and Analgesia, 10.1016/j.vaa.2018.12.009 31178413

[fsn32846-bib-0022] Koster, R. , Anderson, M. , & De Beer, E. (1959). Acetic acid‐induced analgesic screening; 1959: Federation proceedings.

[fsn32846-bib-0023] Kumar, R. S. , Kumar, K. A. , & Murthy, N. V. (2010). Hepatoprotective and antioxidant effects of *Caesalpinia bonducella* on carbon tetrachloride‐induced liver injury in rats. International Research Journal of Plant Science, 1(3), 62–68.

[fsn32846-bib-0024] Lee, J. H. , Johnson, J. V. , & Talcott, S. T. (2005). Identification of ellagic acid conjugates and other polyphenolics in muscadine grapes by HPLC‐ESI‐MS. Journal of Agricultural and Food Chemistry, 53(15), 6003–6010. 10.1021/jf050468r 16028988

[fsn32846-bib-0025] Lima Cavendish, R. , de Souza Santos, J. , Belo Neto, R. , Oliveira Paixao, A. , Valeria Oliveira, J. , Divino de Araujo, E. , Berretta, E. S. A. A. , Maria Thomazzi, S. , Cordeiro Cardoso, J. , & Zanardo Gomes, M. (2015). Antinociceptive and anti‐inflammatory effects of Brazilian red propolis extract and formononetin in rodents. Journal of Ethnopharmacology, 173, 127–133. 10.1016/j.jep.2015.07.022 26192808

[fsn32846-bib-0026] Mansouri, M. T. , Naghizadeh, B. , & Ghorbanzadeh, B. (2015). Ellagic acid enhances the antinociceptive action of venlafaxine in mouse acetic acid‐induced pain: An isobolographic analysis. Pharmacological Reports, 67(3), 473–477. 10.1016/j.pharep.2014.11.004 25933956

[fsn32846-bib-0027] Ojewole, J. A. (2005). Antinociceptive, anti‐inflammatory and antidiabetic effects of *Bryophyllum pinnatum* (Crassulaceae) leaf aqueous extract. Journal of Ethnopharmacology, 99(1), 13–19. 10.1016/j.jep.2005.01.025 15848014

[fsn32846-bib-0028] Pamatz‐Bolaños, T. , Cabrera‐Munguia, D. A. , González, H. , Del Río, R. E. , Rico, J. L. , Rodríguez‐García, G. , Gutiérrez‐Alejandre, A. , Tzompantzi, F. , & Gómez‐Hurtado, M. A. (2018). Transesterification of *Caesalpinia eriostachys* seed oil using heterogeneous and homogeneous basic catalysts. International Journal of Green Energy, 15(8), 465–472. 10.1080/15435075.2018.1473775

[fsn32846-bib-0029] Rao, Y. K. , Fang, S. H. , & Tzeng, Y. M. (2005). Anti‐inflammatory activities of flavonoids isolated from *Caesalpinia pulcherrima* . Journal of Ethnopharmacology, 100(3), 249–253. 10.1016/j.jep.2005.02.039 15893896

[fsn32846-bib-0030] Regalado, A. I. , Mancebo, B. , Paixao, A. , Lopez, Y. , Merino, N. , & Sanchez, L. M. (2017). Antinociceptive activity of methanol extract of *Tabebuia hypoleuca* (C. Wright ex Sauvalle) Urb. Stems. Medical Principles and Practice, 26(4), 368–374. 10.1159/000478015 28591753 PMC5768126

[fsn32846-bib-0031] Saldanha, A. A. , Siqueira, J. M. , Castro, A. H. F. , Matos, N. A. , Klein, A. , Silva, D. B. , Carollo, C. A. , & Soares, A. C. (2017). Peripheral and central antinociceptive effects of the butanolic fraction of *Byrsonima verbascifolia* leaves on nociception‐induced models in mice. Inflammopharmacology, 25(1), 81–90. 10.1007/s10787-016-0300-5 28000084

[fsn32846-bib-0032] Sharma, S. , Khare, S. , Dubey, B. , Joshi, A. , & Jain, A. (2019). Analgesic activity of poly herbal formulation in experimental rats by acetic acid induced writhing test model and Hot plate model. Journal of Drug Delivery and Therapeutics, 9(2‐s), 276–280.

[fsn32846-bib-0033] Taghi Mansouri, M. , Naghizadeh, B. , Ghorbanzadeh, B. , & Farbood, Y. (2013). Central and peripheral antinociceptive effects of ellagic acid in different animal models of pain. European Journal of Pharmacology, 707(1–3), 46–53. 10.1016/j.ejphar.2013.03.031 23528359

[fsn32846-bib-0034] Tewtrakul, S. , Tungcharoen, P. , Sudsai, T. , Karalai, C. , Ponglimanont, C. , & Yodsaoue, O. (2015). Antiinflammatory and wound healing effects of *Caesalpinia sappan* L. Phytotherapy Research, 29(6), 850–856. 10.1002/ptr.5321 25760294

[fsn32846-bib-0035] Tjølsen, A. , Berge, O. G. , Hunskaar, S. , Rosland, J. H. , & Hole, K. (1992). The formalin test: An evaluation of the method. Pain, 51(1), 5–17. 10.1016/0304-3959(92)90003-T 1454405

[fsn32846-bib-0036] Wang, Y. U. , Chen, P. , Tang, C. , Wang, Y. , Li, Y. , & Zhang, H. (2014). Antinociceptive and anti‐inflammatory activities of extract and two isolated flavonoids of *Carthamus tinctorius* L. Journal of Ethnopharmacology, 151(2), 944–950. 10.1016/j.jep.2013.12.003 24333963

